# Safety and efficacy of mesenchymal stem cells in COVID‐19 patients: A systematic review and meta‐analysis

**DOI:** 10.1002/iid3.1000

**Published:** 2023-09-22

**Authors:** Cai Yan, Minjie Hu, Rongjuan Dai

**Affiliations:** ^1^ Xiangtan Central Hospital Department of Infectious diseases Xiangtan Hunan province People's Republic of China; ^2^ The First Affiliated Hospital, Department of Cardiothoracic Surgery, Hengyang Medical School University of South China Hengyang Hunan province People's Republic of China; ^3^ The First Affiliated Hospital, Department of Infectious Diseases, Hengyang Medical School University of South China Hengyang Hunan province People's Republic of China

**Keywords:** clinical improvement, COVID‐19, days to hospital discharge, mesenchymal stem cells, meta‐analysis, mortality

## Abstract

**Background:**

Coronavirus disease‐19 (COVID‐19) is a zoonotic disease that has become a global pandemic. The fast evolution of the COVID‐19 pandemic and persist problems make COVID‐19 highly infectious; publicly accessible literature and other sources of information continue to expand in volume. The mesenchymal stem cells (MSCs) therapy efficacy for COVID‐19 is debatable.

**Objective:**

This systematic review and meta‐analysis (SRMA) aimed to evaluate the usefulness of MSCs in treating COVID‐19.

**Methods:**

Relevant publications were retrieved from databases up to April 30, 2022. In the case of dichotomous data, the 95% confidence intervals (CIs) and pooled risk ratio (RR) were estimated with a random effects model (REM) or fixed effects model (FEM). The pooled mean difference (MD) and 95% CIs were calculated with REM or FEM in continuous data. In the outcomes, studies with insufficient or unusable data were reported descriptively.

**Results:**

A total of eight randomized controlled trials (RCTs) with 464 people were chosen for this SRMA. Relative to the control group, mortality was significantly lower in the MSCs group (RR: 0.66, 95% CI: 0.44, 0.99, *Z* = 2.01, *p* = .04); other secondary outcomes, such as the clinical symptom improvement rate improved in the MSCs group (RR: 1.44, 95% CI: 1.05, 1.99, *Z* = 2.24, *p* = .03), clinical symptom improvement time (MD: −4.01, 95% CI: −6.33, −1.68, *Z* = 3.38, *p* = .0007), C‐reactive protein (CRP) (MD: −39.16, 95% CI: −44.39, −33.94, *Z* = 14.70, *p* < .00001) and days to hospital discharge (MD: −3.83, 95% CI: −6.19, −1.48, *Z* = 3.19, *p* = .001) reduced significantly in MSCs group. However, the adverse reaction incidence did not change significantly.

**Conclusions:**

MSCs are a viable therapy option for COVID‐19 because of their safety and potential efficacy. With no significant adverse effects, MSCs can reduce mortality, clinical symptom improvement time, and days to hospital discharge, improve clinical symptoms, and reduce inflammatory cytokines CRP in COVID‐19. However, further high‐quality clinical studies are required to confirm these results.

## Graphical abstract

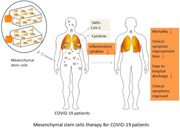

## 1 INTRODUCTION

At the stroke of the New Year 2020, coronavirus disease‐19 (COVID‐19), a zoonotic disease, turned into a global pandemic.^1^ COVID‐19 is caused by infection with severe acute respiratory syndrome coronavirus 2 (SARS‐CoV‐2).^2^ It has been demonstrated that SARS‐CoV‐2 stimulates inflammasomes indirectly or directly, causing pyroptosis and, ultimately, tissue damage and a broad range of clinical symptoms.^3^ COVID‐19 severity must be considered while developing a treatment plan. Patients with mild instances may typically be recovered at home; those with moderate and severe cases should be continuously watched and may need hospitalization; and those with severe and critical cases will have long‐term health impacts from COVID‐19.^2^, ^4^, ^5^, ^6^ Although the COVID‐19 situation is improving, many people may still have contracted the disease and are undergoing treatment. Additionally, there is a possibility of new virus mutations causing a resurgence of the pandemic in the future.^7^, ^8^, ^9^ Therefore, in the event of a second outbreak, finding highly effective treatments for COVID‐19 remains extremely important. Even though other immunotherapies and antiviral agents are still being researched and developed as potential therapies for COVID‐19, only a small number of treatments have been approved for COVID‐19, and their effectiveness in reducing mortality has been limited.^10^, ^11^, ^12^

Recently, one of the most potent therapeutic approaches for COVID‐19 symptoms and complications has been the use of mesenchymal stem cells (MSCs). Moreover, MSCs may help alleviate the long‐term effects of COVID‐19. In particular, MSCs are lauded for their immunomodulatory properties. MSCs were evaluated in treating several diseases.^13^ Human bone marrow cell suspensions were screened to identify MSCs that have lost their hematopoietic potential and form fibroblast cells like proliferating adherent colonies with the ability to differentiate into osteocytes, chondrocytes, and adipocyts both in vivo and in vitro.^14^ Multiple effector functions were modulated when MSCs interacted with immune cells from the adaptive and innate immune systems. MSCs establish peripheral tolerance, migrate to injured tissues, and inhibit proinflammatory cytokine production, promoting damaged cell survival when administered in vivo.^15^ They have substantial benefits for organs associated with COVID‐19.^16^ Previous research has shown that MSCs do not express the SARS‐CoV‐2 spike (S) protein‐recognizing and binding enzymes angiotensin‐converting enzyme 2 (ACE2) or transmembrane serine protease 2 (TMPRSS2). The S protein is crucial to viral infection and transmission.^17^, ^18^ Therefore, MSCs are immune to SARS‐CoV‐2,^19^ and treating COVID‐19 with MSCs might be a successful strategy.

The evolving COVID‐19 pandemic combined with ongoing issues means that systematic review and meta‐analysis are very important. This SRMA will describe the reported performance of MSCs in treating novel coronavirus pneumonia.

## 2 METHODS

### 2.1 Inclusion and exclusion criteria

The following were the inclusion criteria for studies^20^: (1) include COVID‐19 diagnosed patients; (2) only published types of randomized controlled trials (RCTs) were selected; (3) MSCs treatment was the intervention used; (4) the MSCs treatment (intervention arm) was compared with conventional care (control arm); (5) comprehensive information on both groups. Case reports, case series, and review articles were excluded.

### 2.2 Search strategy and study selection

Our SRMA adhered to the Preferred Reporting Items for Systematic Reviews and Meta‐analyses (PRISMA) guidelines for such studies.^21^ The China National Knowledge Infrastructure, the Cochrane Central Register of Controlled Trials (Central), Embase, the Web of Science, and Pubmed, were searched for any mention of COVID‐19 and MSCs until April 30, 2022. Two authors independently checked the abstracts of each article to determine its eligibility.

### 2.3 Study outcome measures

Mortality is the primary outcome, including all deaths that occur between randomization and the endpoint of clinical monitoring. Secondary outcomes include: (1) clinical symptom improvement, including the clinical improvement rate and clinical symptom improvement time; (2) C‐reactive protein (CRP) degree of improvement; (3) incidence of adverse reactions was described as causing pain or discomfort that was not the treatment's intended aim; (4) days to hospital discharge measured from the time of admission till release.

### 2.4 Literature screening, data extraction

The articles that we gathered were managed using Endnote X9. Two authors working independently selected articles no longer supported by the literature searches. After reading the abstract, a full‐text analysis was conducted to check if it met the inclusion criteria. A third author was responsible for resolving any research selection disputes. The arbitrator addressed discrepancies between the data extractions by two independent assessors using a standard data extraction form.^20^ First author, sample size, design, year of publication, country, the intervention of patients in the group (treatment and control group), MSCs dose, and outcomes were the main elements of the extracted data.

### 2.5 Risk of bias (ROB) assessment

The ROB was evaluated using the method described in the Cochrane Handbook for Systematic Reviews of Interventions.^20^ Eligible RCTs had their methodological quality assessed independently. The six types of biases are as follows: (1) Selection bias; (2) Implementation bias; (3) Measurement bias; (4) Follow‐up bias; (5) Reporting bias; (6) Other biases: unbalanced baseline, insufficient sample size, conflict of interest. Each submission was rated as either “low risk,” “unclear risk,” or “high risk”.

### 2.6 Statistical analysis

Stata 16 and RevMan 5.4 were used to conduct the statistical analysis. We utilized pooled data if there were two or more similar studies available.^20^ The risk ratio (RR), 95% confidence interval (CI), and *p* values for dichotomous outcomes were determined. The MD, *p* values, and 95% CI were used for continuous variables. The statistical measure of heterogeneity, *I* ^2^, was used for this analysis. *I* ^2^ < 50% and *p* ≥ .1 indicated that no heterogeneity was present, and *I* ^2^ ≥ 50% or *p <* .1 indicated heterogeneity.^20^ Sensitivity analysis was performed to find the cause of heterogeneity. Publication bias was evaluated using Egger's Test, with a cutoff of *p* < .05 deemed statistically significant.^20^

## 3 RESULTS

### 3.1 Literature selection

**Figure 1:**
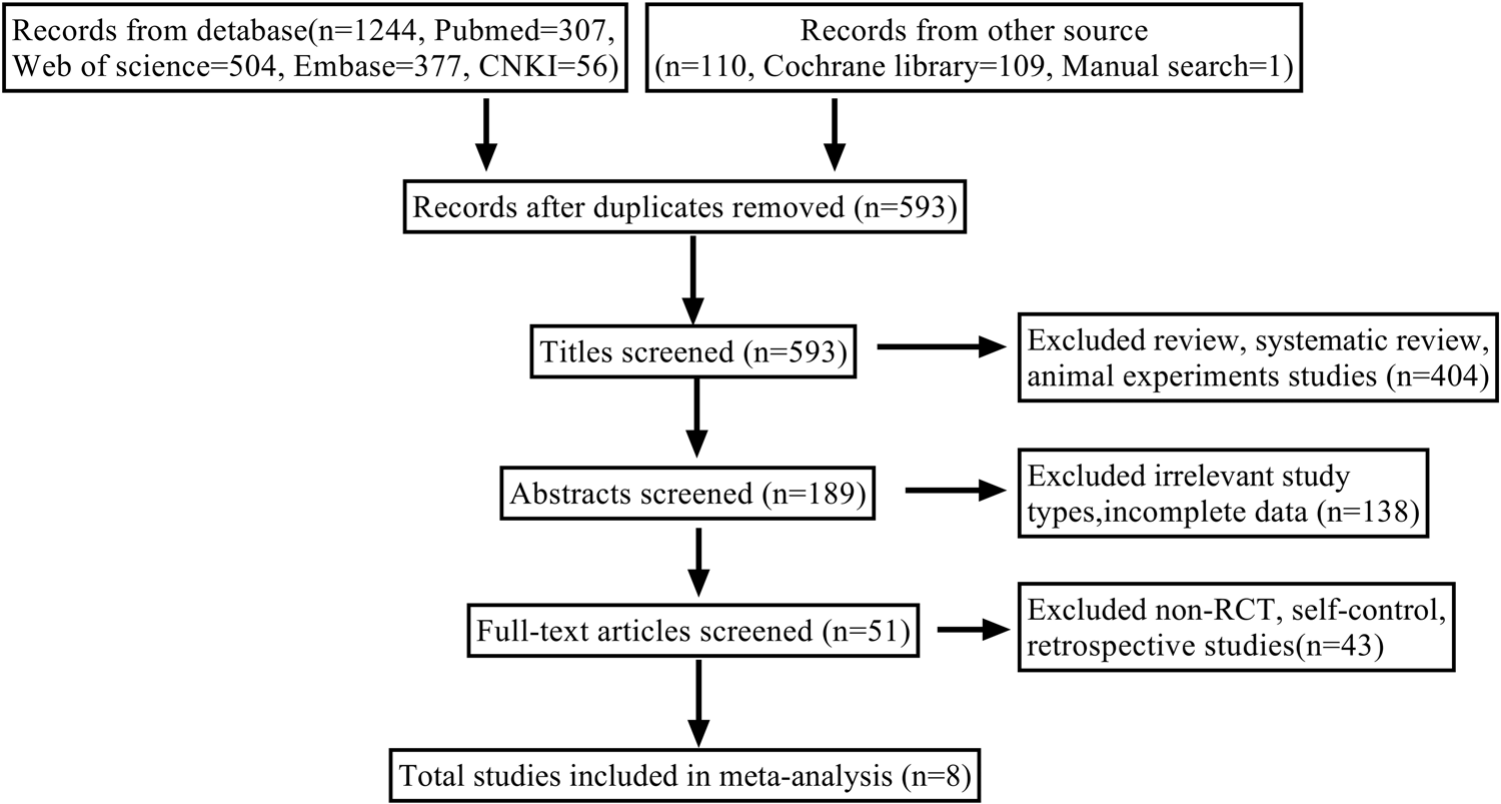
Flow diagram of the number of studies screened and included in the meta‐analysis.

After reviewing the abstracts and titles of the 1354 records obtained by the search strategy, 51 publications were retrieved and analyzed. Only eight RCTs met the criteria for inclusion. Simultaneously, the other articles were ruled out due to their absence of a control group, lack of a randomized controlled trial design, or their status as retrospective studies. Figure 1 is a flowchart depicting the article screening procedure.

### 3.2 Characteristics of the included studies

The details of the eight RCTs that were included are shown in Table 1.^22^, ^23^, ^24^, ^25^, ^26^, ^27^, ^28^, ^29^ There were 464 people at 23 locations, all of whom had been diagnosed with COVID‐19. All articles were written in English and were published between August 2020 and April 2022.

**Table 1:** Characteristics of the included randomized controlled trials.

No.	Author	Country	The publishing year	Design	Sample size (T/C)	Patients enrolled condition	Treatment group	Control group	MSCs dose	Outcomes
1	Yendry Ventura‐Carmenate	The United Arab Emirate	2021	Randomized controlled trial, Open‐label	139(69/70)	Moderate, severe, critically ill	PB‐NHESC‐C + Control group	Standard of care (Favipiravir, Lopinavir/Ritonavir, Enoxaparin, Tocilizumab, Hydroxychloroquine, corticosteroids, antibiotics, antihypertensive and hypoglycemic drugs.)	Compressor (jet) nebulization, 5–6 L/min, total of two doses 24 h apart	1,3,4
2	Lei Shi	China	2021	Randomized controlled Trial, Double‐Blind	100(65/35)	Severe	human UC‐MSCs + standard of care	Placebo + standard of care (respirational support, vasopressor support, renal‐replacement therapy)	4 × 10^7^ cells per infusion on day 0, 3, and 6	4
3	Rongjia Zhu	China	2021	Randomized controlled Trial, Single‐blind	58(29/29)	Common/mild, severe, critically ill	UC‐MSCs+standard of care	Placebo + standard of care (respirational support, corticosteroids, antibiotic, antivirus therapy)	1 × 10^6^ cells/kg	1,2,3,4,5
4	Carmen Lúcia Kuniyoshi Rebelatto	Brazil	2022	Randomized controlled Trial, Double‐Blind	17(11/6)	Critically ill	UC‐MSCs + standard of care	Placebo + standard of care (anticoagulant, steroids, antibiotics)	Three doses of 5 × 10^5^ cells/kg, with a dosing interval of 48–h	1,3,4
5	Antoine Monsel	French	2022	Randomized controlled Trial, Double‐Blind	45(21/24)	Severe, critically ill	UC‐MSCs + standard of care	Placebo + standard of care (corticosteroids)	0.6–1 × 10^6^ cells/kg per dose, 1–3 dose	1,4
6	Lei Shu	China	2022	Randomized controlled trial, Open‐label	41(12/29)	Severe	UC‐MSCs+Control group	Standard treatment (supplemental oxygen, antiviral agents, antibiotic agents, glucocorticoid therapy)	2 × 10^6^ cells/kg	1,2,4,5
7	Giacomo Lanzoni	USA	2021	Randomized controlled Trial, Double‐Blind	24(12/12)	Severe	UC‐MSCs+Control group	Standard of care (heparin, Remdesivir, convalescent plasma, corticosteroids, Tocilizumab, Hydroxychloroquine, Alteplase)+vehicle solution containing human serum albumin and heparin	100 ± 20 × 10^6^ cells each at days 0 and 3	1,4
8	Ismail Hadisoebroto Dilogo	Indonesia	2021	Randomized controlled Trial, Double‐Blind	40(20/20)	Critically ill	UC‐MSCs+Control group	Placebo(100 mL normal saline) + standard of care(Azithromycin, Oseltamivir)	1 × 10^6^ cells/kg	1,4

*Note*: Outcome: 1. mortality rate; 2. clinical improvement rate; 3. CRP; 4. the incidence of adverse reactions; 5. days to hospital discharge.

Abbreviations: C, control group; PB‐NHESC‐C, peripheral blood non‐hematopoieticnonhematopoietic enriched stem cell cocktail; T, treatment group; UC‐MSCs, umbilical cord‐mesenchymal stem cells.

### 3.3 ROB within studies

**Figure 2:**
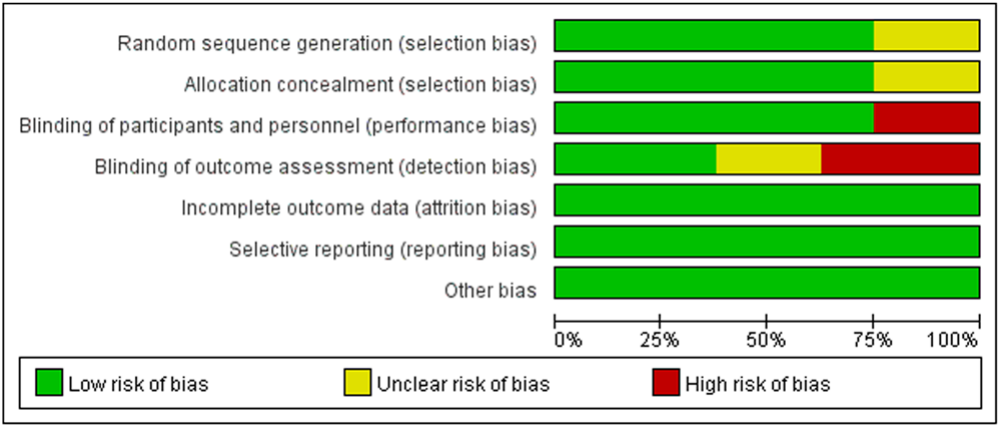
Risk of bias graph: review authors’ judgments about each risk of bias item presented as percentages across all included studies.

**Figure 3:**
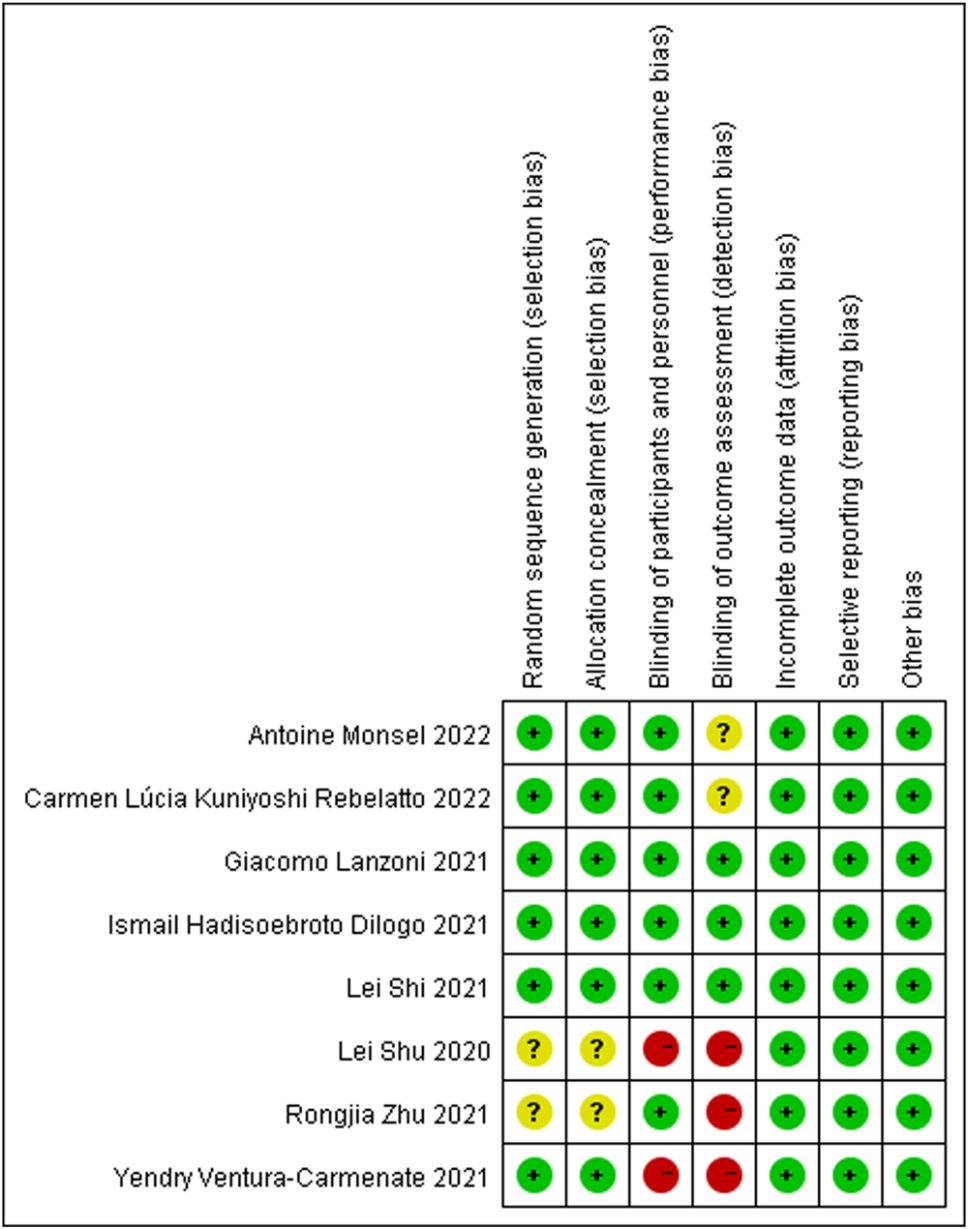
Risk of bias summary: review authors' judgments about each risk of bias item for each included study.

Figure 2 depicts the bias evaluation, while Figure 3 displays the risk summary. Six of the eight RCTs included in this analysis employed computer‐generated randomization, which was considered a low‐risk type.^22^, ^23^, ^25^, ^26^, ^28^, ^29^ The remaining two RCTs were considered to have unclear bias risk because they did not adequately describe their randomization methods.^24^, ^27^ Six studies fully discussed keeping the distribution scheme concealed; this was deemed low‐risk.^22^, ^23^, ^25^, ^26^, ^28^, ^29^ The allocation concealing procedure was not described in the remaining two investigations, which was considered to have an unclear bias risk.^24^, ^27^ The six studies were concealed using the following methods: (1) Randomized block design treatment assignment^22^, ^23^; (2) Randomization table generated by the R program^25^, ^26^; (3) Using SAS software^28^; (4) Using a computerized random number generator.^29^ High risks of detection deviation and performance deviation were found in the three RCTs.^22^, ^24^, ^27^ All eight RCTs were deemed low risk regarding selective reporting of results and data integrity. None of the eight RCTs involved unbalanced baselines, small sample sizes, or conflicts of interest.

### 3.4 Analysis of results

#### 3.4.1 Mortality

**Figure 4:**
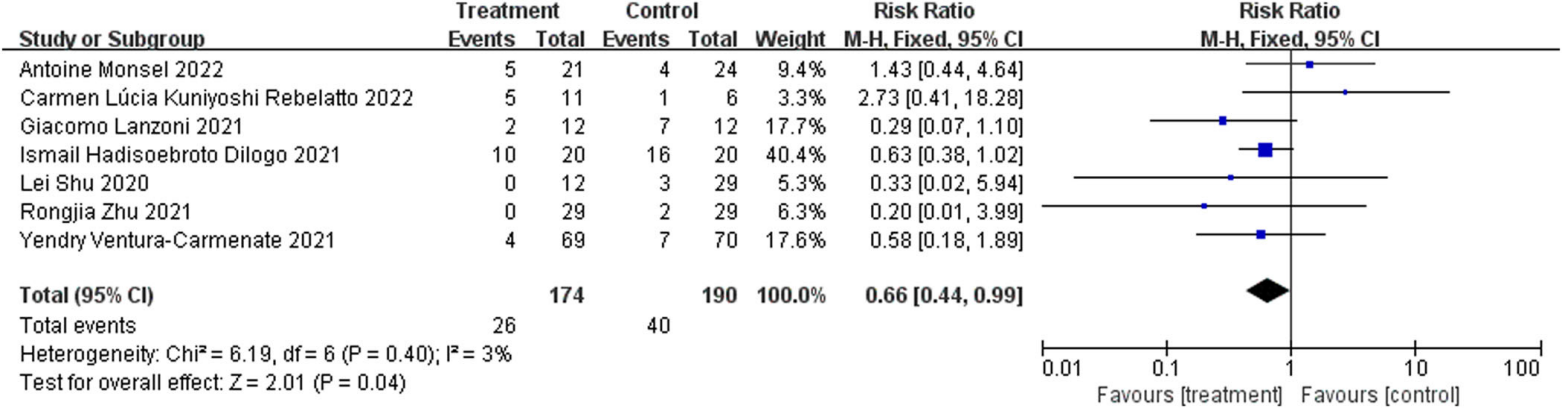
Forest plot of mesenchymal stem cells (MSCs) group and control group with the mortality.

Seven studies^22^, ^24^, ^25^, ^26^, ^27^, ^28^, ^29^ evaluated mortality rates of randomized control group members. The heterogeneity of the included studies was examined, and no heterogeneity was found (*I* ^2^ = 3%, Q test *p* = .4, Figure 4). The fixed effects model (FEM) was implemented. Figure 4 shows a substantial difference across studies (RR: 0.66, 95% CI: 0.44, 0.99, *Z* = 2.01, *p* = .04, Figure 4). The mortality rate was 0.66‐fold among individuals with MSCs compared with those who received either conventional treatment or a placebo. This suggests that MSC‐treated COVID‐19 patients may have an increased chance of survival.

#### 3.4.2 Clinical improvement rate

##### Clinical symptom improvement rate

**Figure 5:**
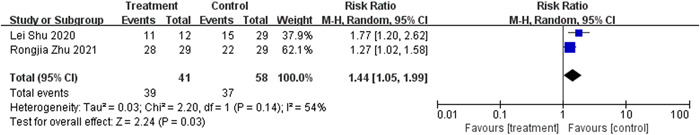
Forest plot of mesenchymal stem cells (MSCs) group and control group with the clinical symptom improvement rate.

There was substantial statistical heterogeneity across the two RCTs^24^, ^27^ that reported improvement of clinical symptoms (*I* ^2^ = 54%, *Q* test *p* = .14, Figure 5); thus, REM was used. Compared with the control group, MSCs showed significantly better results (RR: 1.44, 95% CI: 1.05, 1.99, *Z* = 2.24, *p* = .03, Figure 5). This proved that patients treated with MSCs improved their clinical symptoms at a rate 1.44 times greater than those receiving standard treatment or a placebo. Clinical evidence suggests that MSCs may aid COVID‐19 patients with symptom improvement.

##### Clinical symptom improvement time

**Figure 6:**
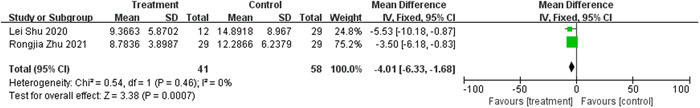
Forest plot of mesenchymal stem cells (MSCs) group and control group with the clinical symptom improvement time.

Two RCTs^24^, ^27^ assessed clinical symptom improvement time and no heterogeneity was found among these studies (*I* ^2^ = 0%, Q test *p* = .46, Figure 6). The FEM was employed, clinical symptom improvement time was substantially shorter in the MSCs group compared with the standard care or placebo group (MD: −4.01, 95% CI: −6.33, −1.68, *Z* = 3.38, *p* = .0007, Figure 6), suggesting that MSCs is effective in reducing clinical symptom improvement time in patients with COVID‐19.

#### 3.4.3 CRP

**Figure 7:**
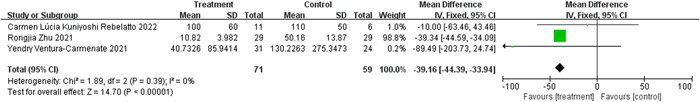
Forest plot of mesenchymal stem cells (MSCs) group and control group with the C‐reactive protein (CRP).

There was no heterogeneity across the studies analyzing CRP levels (*I* ^2^ = 0%, *Q* test *p* = .39, Figure 7) examined in three RCTs.^22^, ^24^, ^25^ The FEM was employed. Participants undergoing MSCs had lower CRP levels than those receiving standard therapy or placebo (MD: −39.16, 95% CI: −44.39, −33.94, *Z* = 14.70, *p* < .00001, Figure 7). MSCs may faciliate CRP reduction in COVID‐19 patients.

#### 3.4.4 Incidence of adverse reactions

**Figure 8:**
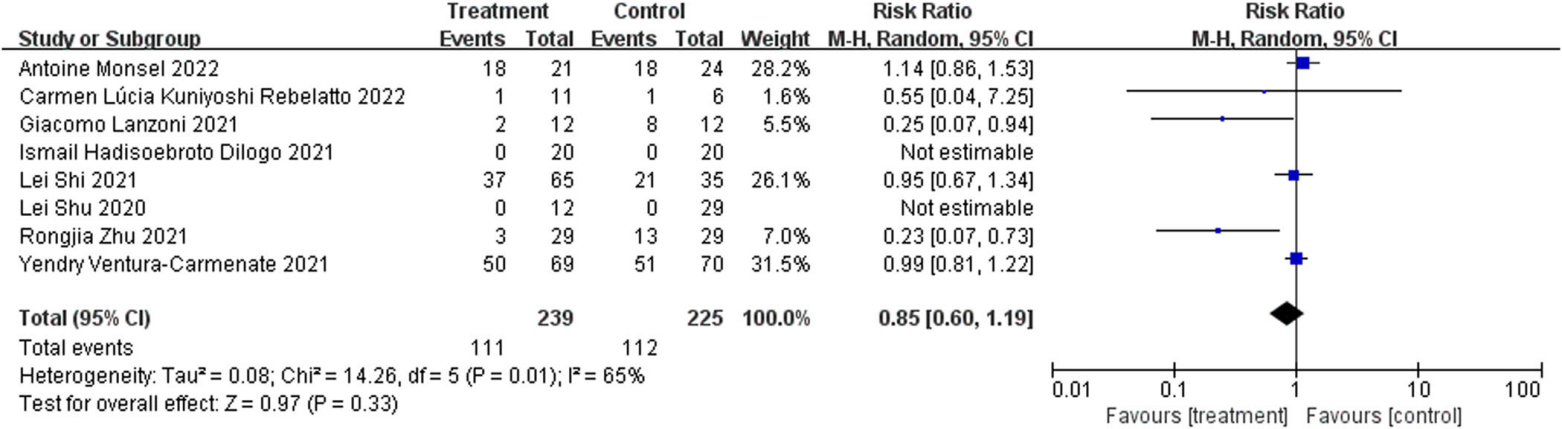
Sensitivity analysis of mesenchymal stem cells (MSCs) group and control group with incidence of adverse reactions.

**Figure 9:**
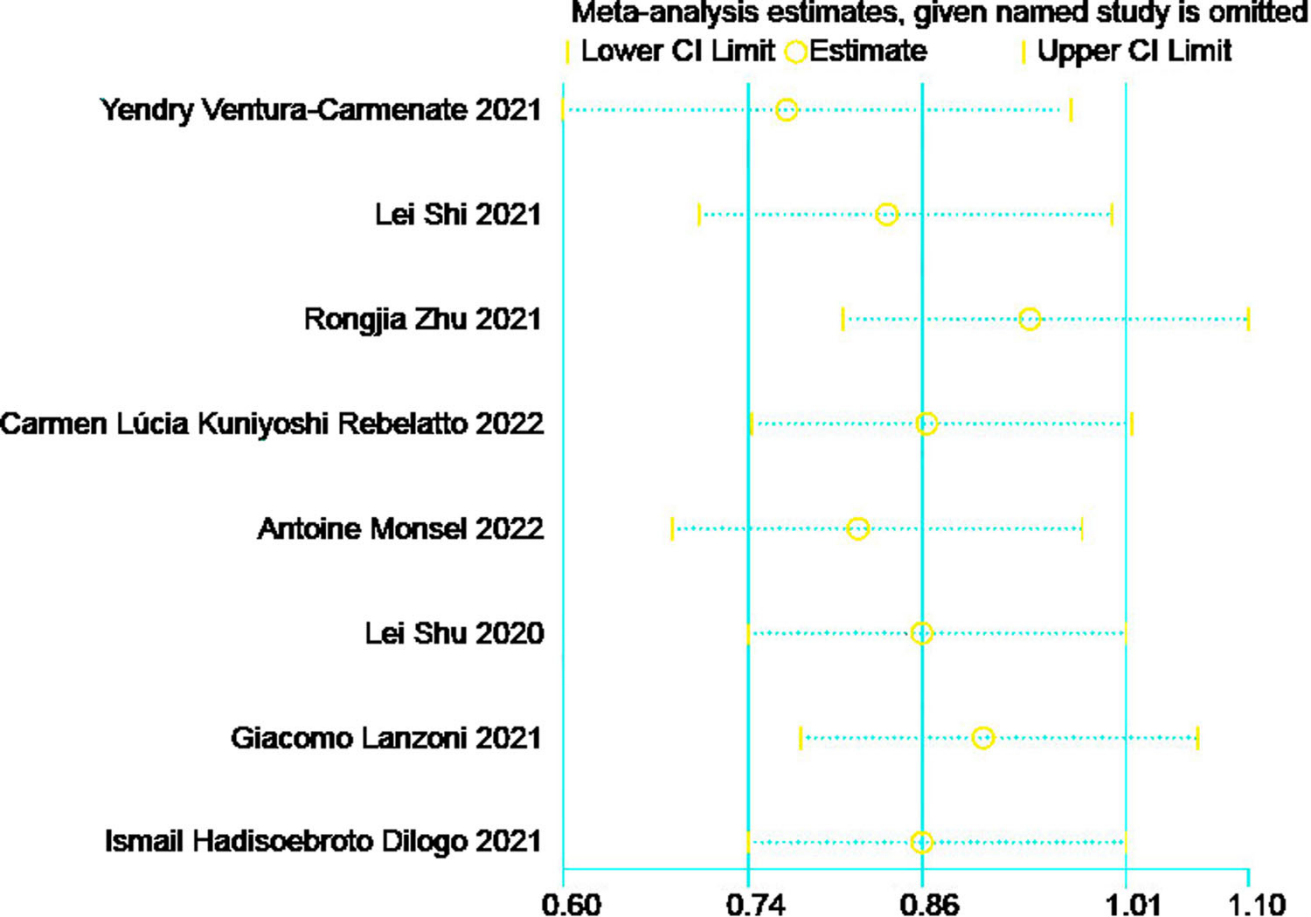
Forest plot of mesenchymal stem cells (MSCs) group and control group with the incidence of adverse reactions.

A statistically significant heterogeneity was found across studies in eight RCTs^22^, ^23^, ^24^, ^25^, ^26^, ^27^, ^28^, ^29^ (*I* ^2^ = 65%, Q test *p* = .01, Figure 8). There was no single research that significantly impacted heterogeneity, as shown by the sensitivity analysis (Figure 9). REM was employed, and no substantial difference was found (RR: 0.85, 95% CI: 0.60, 1.19, *Z* = 0.97, *p* = .33, Figure 8), indicating that MSCs do not impact the incidence of adverse reactions in COVID‐19 patients.

#### 3.4.5 Days to hospital discharge

**Figure 10:**
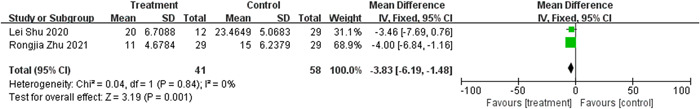
Forest plot of mesenchymal stem cells (MSCs) group and control group with the days to hospital discharge.

There was no significant heterogeneity across the two RCTs^24^, ^27^ regarding the number of days until patients were discharged from the hospital after treatment (*I* ^2^ = 0%, Q test *p* = .84, Figure 10). The FEM was employed. There was a significant difference between the MSCs group and the control group (MD: −3.83, 95% CI: −6.19, −1.48, *Z* = 3.19, *p* = .001, Figure 10), suggesting that patients receiving MSCs were able to leave the hospital sooner than those getting standard care or placebo.

#### 3.4.6 Publication bias detection

**Figure 11:**
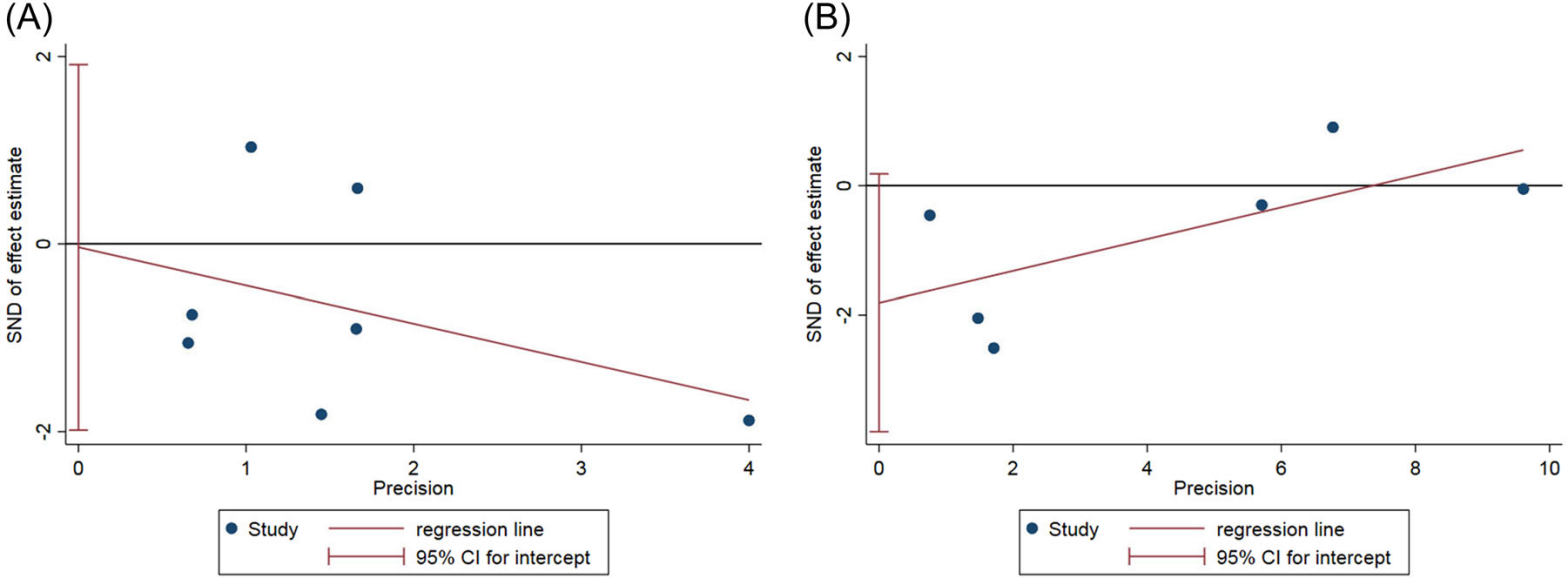
Publication bias (A) mortality; (B) incidence of adverse reactions.

Then, we evaluated meta‐analyses of mortality and adverse effects to see whether they were affected by publication bias. There was no publication bias in terms of mortality and incidence of adverse reactions (*p* > .05; Figure 11A, B); however, the publication bias for other outcomes could not be conducted due to the small number of included publications.

## 4 DISCUSSION

Although the COVID‐19 pandemic is getting better, we still need to seek effective methods for treating COVID‐19 to better respond to this disease in future outbreaks. Thus, research on MSC treatment for COVID‐19 remains significant and worth further exploration. COVID‐19 directly leads to the disordering of adaptive and innate immune responses.^6^, ^30^ Massive vaccination campaigns and antiviral treatment have been implemented, but reinfection and vaccine breakthough instances still occur; thus, researchers have focused on immunotherapies that attempt to lessen the severity of pathological changes in affected organs. In response to various chemokines, MSCs may be recruited to areas of inflammation, where they can regulate the activities of various immunocytes.^31^ Reducing the production of proinflammatory cytokines is one effect of MSCs’ ability to influence host innate and adaptive immune responses.^32^, ^33^ Because of their ability to suppress an overactive inflammatory response, MSCs may be a helpful therapeutic alternative for COVID‐19 patients, especially those in urgent or life‐threatening situations due to lung injury. In fact, from basic studies through human clinical trials, MSC therapy has been a staple of cell‐based treatment.^34^ Multiple clinical trials using intravenous, intratracheal, and inhalation/nebulization administrations of MSC‐based therapeutics are currently registered.^35^ The sources of MSCs are adipose tissue (fat) and bone marrow. Adipose‐derived mesenchymal stem cells (AD‐MSCs) are gaining a lot of attention in the medical field because of their abundant, easy isolation, and potential for various therapeutic applications. The stromal vascular fraction cells (SVFs) provide a rich source of AD‐MSCs.^36^ SVFs and AD‐MSCs present anti‐inflammatory, immune‐modulatory, and proangiogenic activities.^37^ There are some potential uses for AD‐MSCs and SVFs: tissue regeneration and repair and wound healing, autoimmune and inflammatory diseases, cosmetics and beauty apps, cardiovascular disease, neurological Disorders, Hair disease.^38^, ^39^ Acute respiratory distress syndrome (ARDS) patients have also benefited from MSCs treatment.^40^

Results from this meta‐analysis suggest that MSCs treatment may have a therapeutic impact on COVID‐19. In terms of sample size and variety of testing indicators, the current meta‐analysis is the largest RCT research meta‐analyzed of MSCs on COVID‐19 outcome indicators. It evaluated eight RCTs, including MSCs, for the treatment of COVID‐19 providing unambiguous evidence for the efficacy of MSCs. These findings support the hypothesis that MSCs may reduce mortality and hospital stays. Mortality rates were much lower in the MSCs group compared with the control group. CRP was significantly reduced in the MSCs therapy group. CRP is a sensitive biomarker for inflammation and host response to cytokine production.^41^ Regarding clinical manifestation, there was a distinct improvement in clinical symptom improvement time and clinical symptom improvement. These results provide credence to using MSCs therapy to deal with COVID‐19. Furthermore, the results demonstrated that MSCs were generally well‐tolerated by patients and that MSCs therapy was safe, with only minimal side effects.

Patients with COVID‐19 may benefit from MSCs because of their ability to modulate immunity, decrease proinflammatory cytokines, increase plasma antibodies against SARS‐CoV‐2, and promote lung damage repair.^42^, ^43^ Preclinical and clinical studies have demonstrated that MSCs treatment increases the survival rate of individuals with H7N9 influenza.^44^ These results are consistent with those of related research.

There is a dearth of literature on using MSCs in treating COVID‐19 in meta‐analysis. This meta‐analysis has the benefit of including the most RCTs on MSCs therapy for COVID‐19 compared with other published material. All eight publications selected were high‐quality RCTs that did not cherry‐pick their findings and included the complete outcome markers. However, we recognize that there are limitations to our study. First, there is a wide range in sample size, degree of bias, and external validity of the included research. Second, there is a possibility that not all reports on MSC therapy for COVID‐19 may have been included due to the limits of database searches and manual retrieval. Third, there was not enough time between the first publication and the follow‐up study in any of the included articles to observe any long‐term effects. As more evidence becomes available, we will update the literature review. Finally, larger multicohort RCTs with long‐term follow‐up are needed to determine the effectiveness of MSCs treatment in preventing pulmonary fibrosis.

## 5 CONCLUSIONS

MSCs are a viable therapy option for COVID‐19 because of their safety and potential efficacy. MSCs can reduce mortality, clinical symptom improvement time, and days to hospital discharge, with mild adverse effects, improve clinical symptoms, and decrease inflammatory cytokines CRP levels in patients with COVID‐19. Thus, further high‐quality clinical trials are needed to verify these findings.

## AUTHOR CONTRIBUTIONS

**Cai Yan**: Data curation; formal analysis; investigation; methodology; software; validation; writing—original draft; writing—review & editing. **Minjie Hu**: Conceptualization; data curation; supervision; validation; writing—review & editing. **Rongjuan Dai**: Conceptualization; data curation; formal analysis; funding acquisition; methodology; project administration; resources; supervision; writing—review & editing.

## CONFLICT OF INTEREST STATEMENT

The authors declare no conflict of interest.

## Data Availability

All data generated or analyzed during this study are included in this article.
